# Acute aggravation of subdural fluid collection associated with dural metastasis of malignant neoplasms: case report and review of the literature

**DOI:** 10.1007/s10014-013-0162-0

**Published:** 2013-09-15

**Authors:** Shigeyoshi Kimura, Akio Kotani, Toshiro Takimoto, Atsuo Yoshino, Yoichi Katayama

**Affiliations:** 1 Division of Neurosurgery, Department of Neurological Surgery, Nihon University School of Medicine, 30-1 Oyaguchi Kami-cho, Itabashi-ku, Tokyo, 173-8610 Japan; 2 Department of Neurological Surgery, Kasukabe Municipal Hospital, Saitama, Japan; 3 Division of Pathology, Kasukabe Municipal Hospital, Saitama, Japan

**Keywords:** Subdural fluid collection, Dural metastasis, Mucus, Extravasation, Malignant neoplasms

## Abstract

A 63-year-old woman was admitted to our hospital with serious headache and vomiting. Five months before admission, she had undergone surgery for a primary advanced gastric cancer. Neuroradiological examinations revealed subdural fluid collection. We twice performed evacuation of the subdural fluid collection. However, aggravation of her state of consciousness progressed and she passed away. Histological examinations demonstrated that the dural veins were infiltrated by numerous tumor cells that produced mucus; however, ruptured vessels were not found. Furthermore, the subdural fluid collection increased shortly after the initial operation. We infer that the cause of the collection, which was associated with the dural metastasis of malignant tumors, was not only mucin secretion by tumor cells but also a rapid increase in perfusion pressure in the vessels of the dura mater, resulting in extravasation of plasma components into the subdural space. Our case demonstrates that the pathogenetic mechanism that is specific for subdural fluid collection caused by dural metastasis of malignant tumors differs from the mechanism of production of subdural hematoma associated with dural metastasis.

## Introduction

Subdural hematoma and subdural fluid collection are known to occur in association with dural metastasis of malignant tumors that arise outside the central nervous system. However, as regards patients with subdural hematoma caused by dural metastasis of malignant tumors, only about 40 cases have been reported as far as we are aware [1–5, 7–11, 13–19, 21, 23, 25–28, 30–32]. However, there are only five reported cases of subdural fluid collection caused by dural metastasis of malignant tumors [6, 12, 20, 24, 29]. Furthermore, when subdural fluid collection caused by dural metastasis of malignant tumors occurred, it was due to mucous effusion produced by the tumor cells in three of these five patients [6, 12, 20], although none of those three cases underwent histopathological examinations. In our case report, we demonstrate that the pathogenetic mechanism of the subdural fluid collection associated with dural metastasis differed from that of subdural hematomas associated with dural metastasis reported previously.

## Case report

**Fig. 1:**
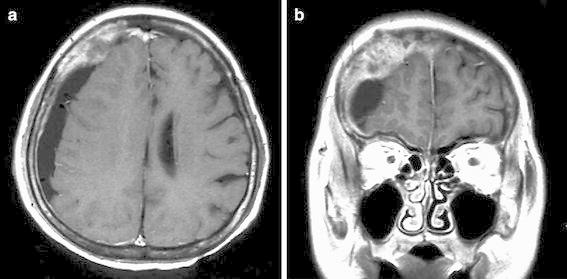
T1-weighted magnetic resonance with gadolinium (**a** horizontal section, **b** coronal section), revealing subdural fluid collection on the right side, with a tumor growing into the subdural space and invading the skull bone and dura mater. However, no other metastatic lesions were observed in the brain and meninges

A 63-year-old woman was admitted to our hospital with serious headache and vomiting. Five months before admission, she had undergone surgery for a primary advanced gastric cancer at a local hospital. However, as the hospital went bankrupt, it was impossible for us to obtain details of her histopathological information. On admission, her consciousness level was Glasgow Coma Scale score 14 (E4, V4, M5). Her arms and legs showed no paralysis, and there was no history of head trauma. Blood examinations revealed mild anemia and a decrease in platelet count. However, this did not represent a state of disseminated intravascular coagulation (DIC). Head computed tomography (CT) revealed a crescent-shaped fluid collection in the right frontoparietal subdural space with a solid mass attached to the internal surface of the cranial bone. Similarly, magnetic resonance imaging (MRI) showed a tumor that projected into the subdural space and invaded the skull bone and dura mater (Fig. 1a, b). However, no metastatic lesions were observed in the brain and meninges. Local recurrence of gastric cancer and metastases were not confirmed. In addition, the presence of breast cancer, lung cancer, and gastrointestinal carcinoma was excluded based on a systemic search. We diagnosed metastasis from gastric cancer to the dura mater complicated by subdural fluid collection. However, the precise relationship between tumor and subdural fluid collection remained unclear.

At the first operation, burr-hole evacuation of the subdural fluid collection was performed under local anesthesia. The membrane was not present under the dura mater. The subdural fluid collection displayed a xanthochromic yellow transparence with viscosity. Furthermore, it solidified immediately at room temperature, compatible with Froin syndrome, and there was no evidence of hemorrhage. Cytological examinations revealed no blood cells or malignant neoplastic cells within the fluid collection. Although symptoms improved temporarily, serious headache reappeared, and disturbance of consciousness developed 2 days later. Head CT again demonstrated an increase in subdural fluid collection. Although the patient’s illness was already in its terminal phase, her family expected symptoms to improve. We therefore performed craniotomy under general anesthesia 3 weeks after the first operation in order to prevent recurrence of the subdural fluid collection and tumor removal.

At the second operation, the tumor was found to be growing in the periosteum, muscle, and subcutaneous tissue through the cranial bone, wide invasion of the tumor into the dura mater under the craniotomy was evident. The membrane was not observed in the subdural space. The subdural fluid collection displayed a yellowish transparence similar to that at the first operation. The tumor had not invaded the brain and meninges.

**Fig. 2:**
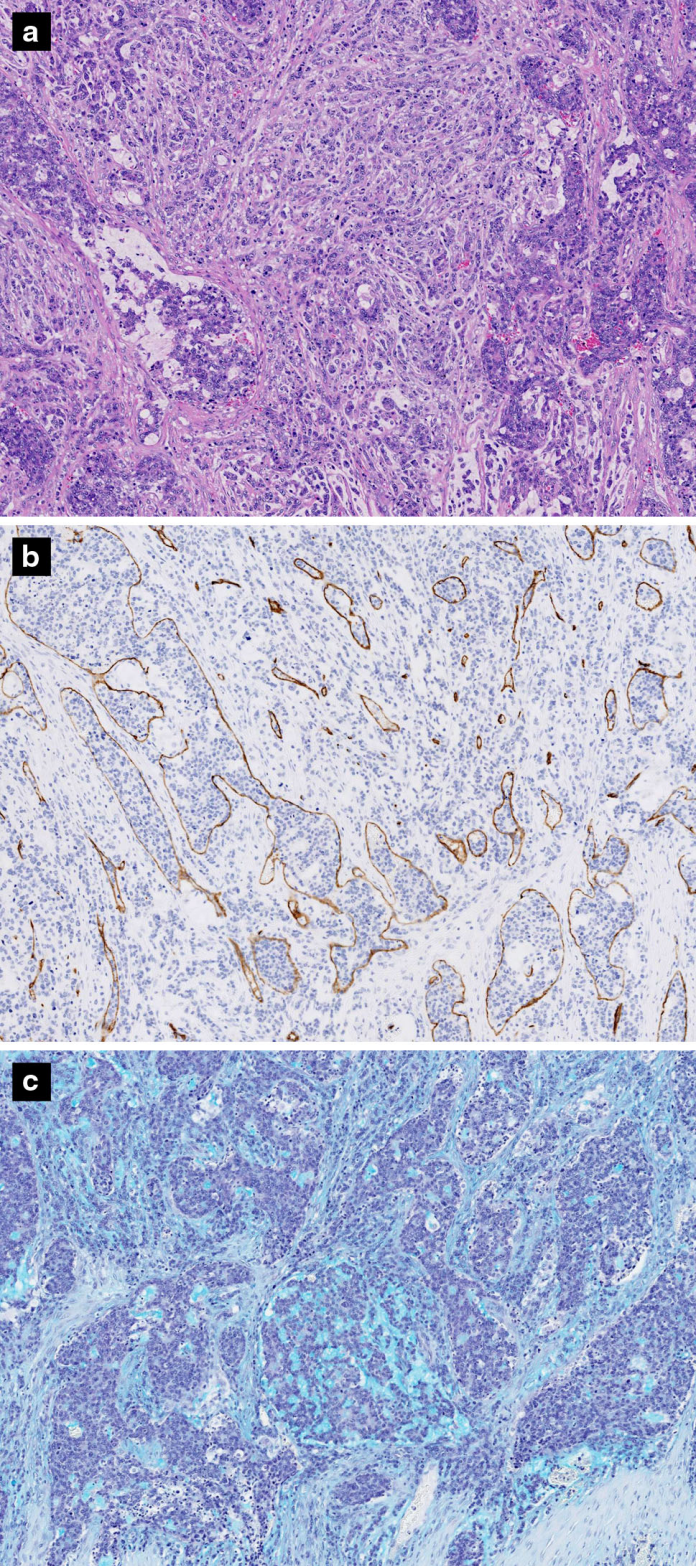
**a** Microscopic views of the dura mater on hematoxylin and eosin staining: Poorly differentiated adenocarcinoma cells diffusely invade the dura mater. However, ruptured vessels and leakage of blood corpuscle components around the vessels are not observed (×50). **b** Microscopic views of the dura mater on CD34 immunostaining: tumor cells infiltrating and occluding multiple vessels (×50). **c** Alcian blue staining: numerous tumor cells are positive intracellularly; furthermore, mucinous material was observed in the stroma (×50)

Histological examinations of the dura mater revealed diffuse infiltration by malignant neoplastic cells. These cells exhibited a cribriform pattern and tubular formation, compatible with a diagnosis of metastatic, poorly differentiated adenocarcinoma of the dura mater (Fig. 2a). We were unable to compare this specimen with the gastric adenocarcinoma found at the previous hospital. However, we reached the definitive diagnosis of metastasis from the primary gastric cancer to the dura mater, because no malignant tumors in other organs were confirmed. Almost all vessels of the dura mater were infiltrated by numerous tumor cells (Fig. 2b). However, ruptured vessels and leakage of blood corpuscle components around the vessels were not found (Fig. 2a). On special staining, many tumor cells demonstrated positivity for Alcian blue, which is compatible with the presence of adenocarcinoma cells that produce mucus. Furthermore, mucinous material was observed in the stroma (Fig. 2c). These findings strongly suggested that the subdural fluid collection was a consequence of mucus secretion by the adenocarcinoma. After the second operation, the patient’s headache improved, but aggravation of her state of consciousness progressed again and she fell into a coma 2 weeks later. She died of DIC on day 78 after hospitalization. Permission for autopsy was not obtained.

## Discussion

Nontraumatic subdural fluid collection associated with dural metastasis is quite rare. To our knowledge, only five cases [6, 13, 20, 24, 29] have so far been described (Table 1), and in the previous reports, no mucous material was investigated by histopathological examinations.

**Table 1:** Summary of cases of subdural fluid collection associated with dural metastasis of malignant neoplasms arising in other organs

Authors	Age (years), sex	Primary tumor site	Histological diagnosis	Blood cells in subdural fluid collection	Ruptured vessels and leakage of blood cells in dura mater	Treatment	Outcome (survival time)
McDonald et al. [20]	43, male	Cutaneous (clinical diagnosis)	Hodgkin’s disease	None	None	Burrhole irrigation	Dead (2 months)
Castleman et al. [6]	71, male	Pancreas	Adenocarcinoma	None	None	Conservative	Dead (1 month)
Rao et al. [24]	60, male	Unknown (bronchogenic?)	Anaplastic carcinoma (not described)	(Not examined)	None	Conservative	Dead (3 months)
Tasaki et al. [29]	61, male	Prostate	Adenocarcinoma	None	None	Burrhole irrigation, craniotomy, chemotherapy	Dead (6 months)
Kamada et al. [12]	63, male	Rectum	Adenocarcinoma	None	None	Burrhole irrigation, craniotomy	Dead (3 months)
Our case	63, female	Stomach	Adenocarcinoma	None	None	Burrhole irrigation, craniotomy	Dead (78 days)

In past reports, the pathogenetic mechanism of subdural hematoma caused by dural metastasis of malignant tumors had been described, as follows. The dura mater is composed of two layers: a dense outer layer and a loose areolar inner layer of the leptomeningeal surface of the dura mater. The veins of the areolar inner layer drain into the veins of the dense outer layer and finally into the periosteal venous system [15, 19, 24, 26]. Metastasis of malignant tumors to the dura mater secondary to spread from the adjacent bone is most common [19, 22, 25, 27, 29]. In addition, tumor cells spread by retrograde dissemination through the connecting channels of the venous system between the diploic veins in the cranial bone and the dura mater [15, 19, 22, 27] or through the perivertebral venous plexus (Batson’s plexus) [11, 29]. Russell and Cairns considered that obstruction by metastatic tumor cells of the draining veins in the dense outer layer increased retrograde pressure of proximal capillaries in the areolar inner layer, resulting in dilation and rupture of capillaries and subsequent subdural hematoma [26]. However, our patient did not have blood coagulation disorders, and the subdural fluid collection displayed a xanthochromic yellow transparence with viscosity; Froin syndrome was also present. Moreover, cytological examinations revealed no blood cells or neoplastic cells in the fluid collection. On this basis, our case is considered to be different from subdural hematoma associated with dural metastasis. In our case, histological examinations showed that the vessels of the dura mater were infiltrated by numerous tumor cells that showed positive for mucus production on Alcian blue staining. However, no ruptured vessels were found. We presumed that metastatic adenocarcinoma of the dura mater secreted mucus causing the subdural fluid collection. Nevertheless, as we encountered re-enlargement of the subdural fluid collection shortly after the initial operation, it seems unlikely that fluid secretion by tumor cells alone could have caused the rapid increase in subdural fluid collection. Kamada et al. [12] indicated that subdural fluid collection has been inferred to occur as a result of secretion by the tumor cells themselves and extravasation of plasma components caused by an increase in capillary transmural pressure. Similarly, in our case, it seems that subdural fluid collection was caused by fluid secretion from tumor cells combined with a rapid increase in perfusion pressure in capillaries of the areolar inner layer, resulting in extravasation of plasma components into the subdural space.

The primary sites of tumors that cause subdural fluid collection following dural metastasis of malignant tumors are widely divergent, and involve Hodgkin’s disease [20], pancreatic cancer [6], prostate cancer [29], and rectal cancer [12] (Table 1). On the other hand, primary sites of tumors giving rise to subdural hematoma associated with dural metastasis are gastric, prostate, pancreatic, breast, lung, and uterine cancers [10, 11, 13, 17–19, 22, 23, 29]. Adenocarcinoma is the most common histological diagnosis for subdural hematoma caused by dural metastasis of malignant tumors [11, 13, 17, 28, 29]. Similarly, three of the five reported patients [6, 12, 29] demonstrating subdural fluid collection associated with dural metastasis had adenocarcinoma (Table 1).

The prognosis of subdural fluid collection caused by dural metastasis of malignant tumors is extremely poor and the mortality rate is high, because all reported patients with subdural fluid collection died within a short period [6, 12, 20, 24, 29] (Table 1). It is expected, therefore, that the effect of surgical management of such cases may not result in improvement. On the other hand, aggressive surgical treatments can achieve a favorable outcome in some cases [3–5, 7, 10, 25], so that the therapeutic approach employed should be chosen according to the individual condition of each patient. When the overall status of the patient is poor, insertion of an Ommaya reservoir may be effective [11, 13]; postoperative irradiation appears to be necessary to control the residual tumor [2, 11, 17]. However, whether or not radiation therapy can prevent relapse remains questionable.

We conclude that although subdural hematoma and subdural fluid collection caused by dural metastasis of malignant tumors exist in a similar state, the case presented in our report is important in that it shows that, in actuality, each may occur through different pathogenetic mechanisms. We consider that the etiology of subdural fluid collection caused by dural metastasis of malignant tumors involves not only fluid secretion by tumor cells of the dura mater but also by a rapid increase in perfusion pressure in capillaries of the areolar inner layer, resulting in extravasation of plasma components into the subdural space.
